# Elevated triglycerides level in hospital stay as a risk factor of mortality in patients with severe acute pancreatitis

**DOI:** 10.1371/journal.pone.0207875

**Published:** 2018-11-29

**Authors:** Qin Wu, Min Fu, Kexin Zheng, Hong Bo, Hao Yang, Xi Zhong, Guanlin Liang, Yujun Xu, Bibo Hao, Zhi Hu, Zhongwei Zhang, Xiaodong Jin, Yan Kang

**Affiliations:** 1 Department of Critical Care Medicine, West China Hospital, Sichuan University, Chengdu, China; 2 IBM Research, China, Beijing, China; University of Szeged, HUNGARY

## Abstract

Hypertriglyceridaemia is one of the most common causes of severe acute pancreatitis (SAP). However, the association between elevated triglycerides (TG) level in hospital stay and outcome in SAP patients with normal TG level at admission has not been clearly demonstrated. This retrospective study assessed the serum TG levels of patients with SAP admitted to the intensive care unit (ICU) in 2017. Variables with a statistically significant association with the incidence of in-hospital TG elevation, as determined by univariate analysis, were analysed using a logistic regression model to predictors. Of the 99 patients included in the study, TG levels were within the normal range in 59 (59.59%) patients at admission. Among patients with normal TG level when admitted to ICU, 28 (47.46%) experienced at least one episode of TG level elevation during their ICU stay. Elevated TG level in hospitalization is associated with an increased length of ICU stay, as well as increased mortality. In addition to other factors, propofol usage was independently associated with the occurrence of in-hospital-TG elevation. To conclude, we retrospectively investigated the incidence, outcome, and risk factors for in-hospital TG elevation events in SAP patients admitted to the ICU. We found a high incidence of both preexisting and in-hospital-acquired TG elevation in SAP patients admitted to the ICU. The TG elevation that occurred during the ICU stay was associated with worse outcomes and long-term hospitalization of the ICU. Propofol usage was independently associated with the TG elevation occurrence in the ICU.

## Introduction

Acute pancreatitis, which is sudden inflammation of the pancreas, is usually a mild disease with a favourable outcome [1, 2]. About 20% of patients with acute pancreatitis develop moderately severe or severe acute pancreatitis (SAP) (as defined by the revised Atlanta classification), which can be life-threatening [3]. Although the overall mortality rate for acute pancreatitis is relatively low, the mortality rate for SAP is much higher, at 10% for patients with sterile pancreatic necrosis and 25% for patients with infected pancreatic necrosis, compared with mild acute pancreatitis, which has a mortality rate of less than 1% [1, 4]. Because of organ dysfunction, patients with SAP may be hospitalised for more than 2 weeks and frequently require a stay in the intensive care unit (ICU) [5]. Of the approximately 210,000 patients admitted to hospitals in the United States each year with acute pancreatitis, about 20% have SAP[6]. Moreover, the incidence of SAP in Europe and the United States is increasing by about 5% per year, mainly owing to an increase in biliary pancreatitis [7, 8].

The inflammatory reaction that occurs in SAP is thought to be due to premature activation of pancreatic proteases, which promote acinar cell apoptosis and necrosis [9]. Pancreatic inflammation disrupts pancreatic lipase secretion, which impairs triglyceride (TG) absorption by the intestinal mucosa cells. The unabsorbed TGs are then hydrolysed by pancreatic lipase, generating free fatty acids and 2-monoglycerides, which leads to dyslipidaemia [10]. Many studies have focused on the mechanism by which hypertriglyceridaemia triggers SAP [11]. However, the dynamic changes in serum TG levels and the impact of TG level elevation in SAP, especially the new-onset during an ICU stay, are poorly understood. This prompted us to investigate whether changes in serum TG levels over time are associated with SAP outcomes.

## Methods

### Study population

This single-centre retrospective study was conducted in the Department of Critical Care Medicine, West China Hospital, Sichuan, China. The clinical research ethics boards of the West China Hospital approved the study and waived the need for participants’ informed consent because of the study’s retrospective, anonymous, and non-interventional nature. All methods were performed in accordance with the relevant guidelines and regulations. We retrospectively analysed data via individual chart review from all patients admitted with pancreatitis to the Department of Critical Care Medicine between January 2017 to December 2017 using a prospectively collected hospital database. A specialised system (Clinical Information System designed for Critical Care Practitioners, Sichuan Smart Medicine Medical Information System Company, Chengdu, China) was used to ensure data integrity.

### Study plan

The diagnosis of acute pancreatitis is based on symptoms, physical examination and laboratory selection values and requires two of the following three characteristics: 1) acute onset of upper abdominal pain, 2) serum amylase or lipase activity greater than 3 times the normal level, and 3) findings on cross-sectional abdominal imaging consistent with acute pancreatitis [12]. All patients admitted to the ICU during the study period with a diagnosis of pancreatitis were identified by scanning the medical records. SAP was defined as acute pancreatitis with persistent organ failure lasting for more than 48 h. Exclusion criteria included any of the following: age younger than 18 years, ICU stays less than 24 h, recurrence of chronic pancreatitis, a time from abdominal pain onset to hospital admission of more than 72 h, mild acute pancreatitis, and unavailable laboratory measurements or medical records. For patients with more than one ICU stay during the study period, only the first episode was analysed.

Gallstone SAP was diagnosed if imaging studies (computed tomography, magnetic resonance imaging, or ultrasonography) showed gallstones or choledocholithiasis. Hyperlipidemic SAP was defined as TG levels greater than 11.3 mmol/L or TG levels greater than 5.65 mmol/L with grossly lipaemic serum. Alcoholic SAP was diagnosed if the patient had a history of heavy alcohol consumption before the SAP onset and a history of alcohol consumption of over 50 g/d for longer than 5 years.

For the purposes of this study, TG elevation event was defined when a serum TG level > 1.70 mmol/L was detected. The patient population was divided into two groups based on serum TG level at admission: a normal TG level group (N-TG group) and an elevated TG level group (E-TG group). Serum TG levels during the ICU stay were reviewed to rule out any episodes of new-onset TG elevation among patients with a normal TG level when admitted to ICU. Patients in the N-TG group were included in the in-hospital TG elevation group (H-E-TG group) if they experienced any episode of TG elevation while hospitalised, whereas members of in-hospital normal TG level group (H-N-TG group) did not experience any TG elevation episodes.

### Data collection

Venous blood for all laboratory tests (including TG) was drawn within 6 h after admission and again whenever required, based on the judgement of the physician. Arterial blood was collected for air blood gas analysis whenever necessary. Specifically, TG was measured at the Department of Laboratory Medicines of West China Hospital using a Biochemical Analyzer (Dimension AR/AVL Clinical Chemistry System, Newark, NJ, USA). Other laboratory parameters were collected at admission and during hospitalisation, including: prothrombin time, aspartate aminotransferase, total protein, red blood cell count, haemoglobin level, direct bilirubin, arterial carbon dioxide partial pressure, haematocrit, activated partial thromboplastin time, mean corpuscular volume, mean corpuscular haemoglobin, high-density lipoprotein cholesterol, fibrinogen, mean corpuscular haemoglobin concentration, red blood cell distribution width, platelet count, white blood cell count, international normalized ratio, thrombin time, arterial oxygen partial pressure, base excess, lactate, procalcitonin, total bilirubin, alanine aminotransferase, albumin, glucose, cystatin C, gamma-glutamyl transferase, low-density lipoprotein cholesterol alkaline phosphatase and TG. Data routinely recorded on admission included age, gender, aetiology, and disease severity using the Acute Physiology and Chronic Health Evaluation II (APACHE II) score and Ranson’s score at admission.

### Statistical analyses

The data were analysed using SPSS Statistics 17 (SPSS Software, Chicago, IL, USA) and GraphPad Prism (GraphPad Software, San Diego, CA, USA) software. Continuous variables are presented as means ± standard deviation or median and interquartile range, while categorical variables are expressed as percentages. Analysis of variance (ANOVA) tests were used to compare means for continuous variables. Fisher's exact test was used for categorical variables. Chi-square tests were used to compare proportions for categorical variables. The log-rank test was used to evaluate statistical differences in survival curves, which were constructed using the Kaplan-Meier method. Univariate and multivariate logistic regression analyses were used to identify variables predictive of TG elevation event. A p value of <0.05 indicated a significant difference.

## Results

**Fig 1:**
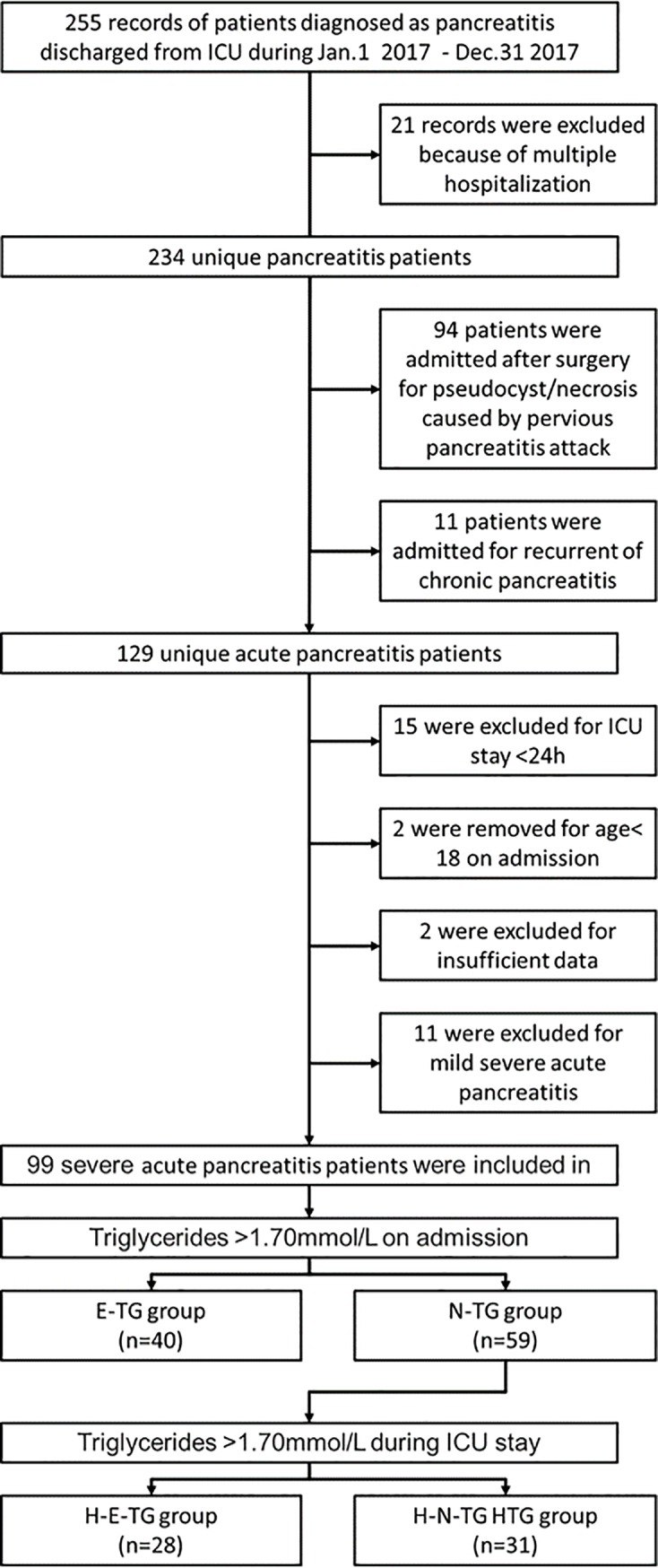
Flowchart of current study. A total of 234 unique pancreatitis patients was retrospectively collected and 2 of them were excluded for their age was less than 18 years at admission. Among them, 99 patients were diagnosed with severe acute pancreatitis and stayed in the ICU for more than 24 h. For enrolled SAP patients, 40 were admitted with an elevated TG level at admission. The remaining 59 patients with a normal TG level, 28 experienced at least one episode of TG elevation event during hospitalization.

We identified 255 adult ICU patients with a diagnosis of pancreatitis during the study period. Twenty-one patients were excluded because they were readmitted to the ICU during the study period. One hundred and twenty-nine patients who met the diagnosis of acute pancreatitis were enrolled after patients who were admitted because of recurrent chronic pancreatitis were excluded. Moreover, 15 whose ICU stay was < 24 h and 11 who had only mild acute pancreatitis were excluded, as well as two who were aged < 18 years at admission and two with insufficient data. Thus, 99 patients admitted for SAP were ultimately included in the study. Serum TG at the time of admission was higher than 1.70 mmol/L in 40 patients, who were therefore categorised as E-TG group. Thirty-one patients with normal TG level at admission experienced at least one episode of TG elevation during their ICU stay, and these patients were included in the H-E-TG group (Fig 1).

**Fig 2:**
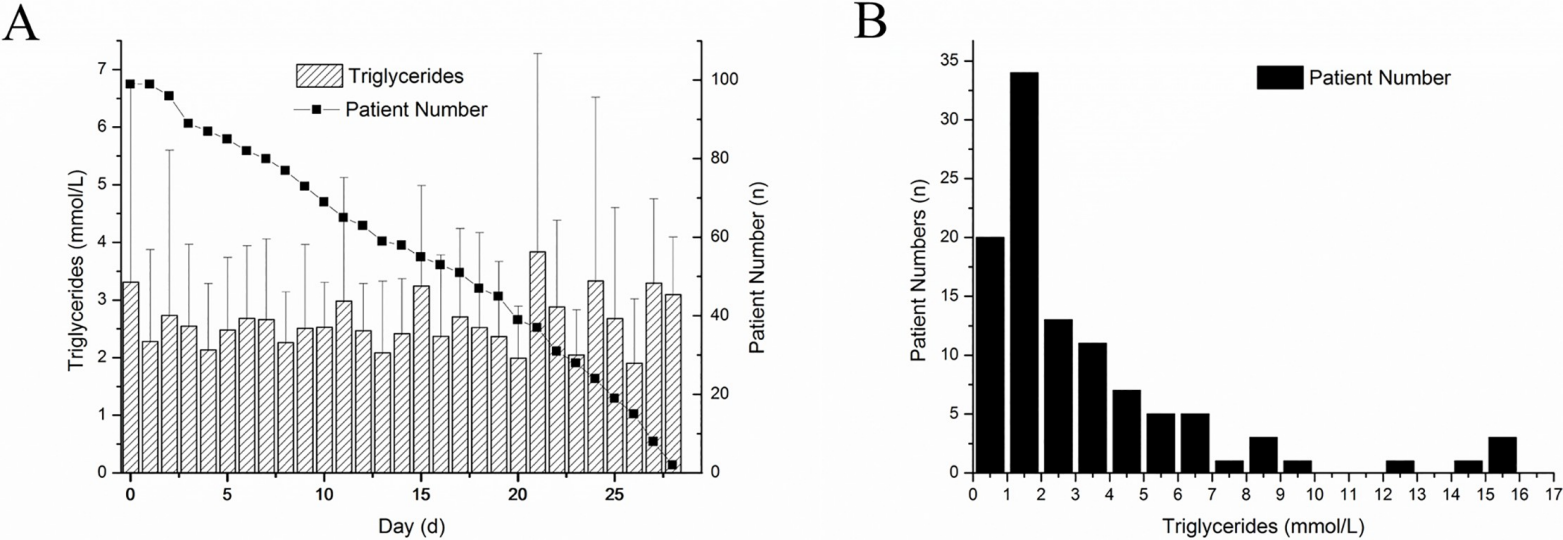
A: Changing trend of TG level from Day 0 to Day 28. Bar chart representing average TG level and patient numbers after admission. Day 0 stands for the admission. B: Distribution of TG level at admission for all enrolled patients. Forty SAP patients were admitted with an elevated TG level.

The baseline characteristics of the study population are summarised in Table 1 and S1 Table. Gallstone SAP was the most common cause for SAP in this cohort. Mean APACHE II and Ranson’s scores were 16.72 and 2.13, respectively. In addition, overall in-hospital mortality was 28.28%, and 28-day mortality was 21.21%. The average serum TG levels in the study cohort for 28 days after ICU admission and the distribution of serum TG levels in all patients upon admission are shown in Fig 2.

**Table 1:** Demographics, clinical and outcome data of SAP patient cohort.

Parameters	All (n = 99)	N-TG Group (n = 59)	E-TG Group (n = 40)	P
Demographic Data				
Age, mean (SD),y	48.71 (13.97)	50.92 (15.20)	45.45 (11.35)	0.056
Male, n (%)	65 (65.66)	36 (61.02)	29 (72.50)	0.238
Etiology, n (%)				<0.001
Gallstone	43 (43.43)	36 (61.02)	7 (17.50)	
Hyperlipidemia	19 (19.19)	0 (0.00)	19 (47.50)	
Alcoholic	6 (6.06)	0 (0.00)	6 (15.00)	
Others	31 (31.32)	23 (38.98)	8 (20.00)	
Underlying medical conditions				
Hypertension, n (%)	22 (22.22)	15 (25.40)	7 (17.50)	0.357
Diabetes Mellitus, n (%)	20 (20.20)	12 (20.34)	8 (20.00)	0.968
APACHEⅡScore*, mean (SD)	16.72 (8.11)	15.85 (8.58)	18.05 (7.28)	0.191
Ranson’s Score*, mean (SD)	2.13 (1.13)	1.93 (1.13)	2.43 (1.08)	0.033
TG, mean (SD), mmol/L	3.29 (3.44)	1.26 (2.49)	6.12 (3.79)	<0.001
TG, mean (SD), mg/dL	291.16 (304.44)	111.51 (220.36)	541.62 (335.42)	<0.001
CHOL, mean (SD), mmol/L	3.20 (2.55)	1.88 (0.64)	5.02 (3.04)	<0.001
HDL-C, mean (SD), mmol/L	0.39 (0.32)	0.35 (0.21)	0.44 (0.42)	0.165
LDL-C, mean (SD), mmol/L	0.91 (0.69)	0.84 (0.50)	1.02 (0.89)	0.203
Mechanical Ventilation free days^#^, mean (SD), d	19.28 (341.65)	20.07 (8.17)	18.13 (9.08)	0.732
Renal replacement therapy, n (%)	8 (7.90)	5 (8.50)	3 (7.50)	0.863
Time to start enteral nutrition, mean (SD), d	1.90 (2.99)	1.57 (2.15)	2.35 (3.86)	0.251
Time for parenteral nutrition^#^, mean (SD), d	9.16 (8.24)	8.19 (7.63)	10.60 (8.96)	0.153
28-day mortality, n (%)	21 (21.21)	15 (25.42)	6 (15.00)	0.213
In hospital mortality, n (%)	28 (28.28)	19 (32.20)	9 (22.50)	0.293
ICU LOS, mean (SD),d	16.80 (16.10)	14.32 (12.36)	20.35 (19.91)	0.314

APACHE: Acute Physiology and Chronic Health Evaluation; TG: Triglycerides, CHOL: cholesterol, HDL-C: High-density lipoprotein cholesterol, LDL-C: Low-density lipoprotein cholesterol, H/L: HDL-C/ LDL-C, LOS: length of stay; IQR: interquartile range

*on admission

^#^within 28 days

**Fig 3:**
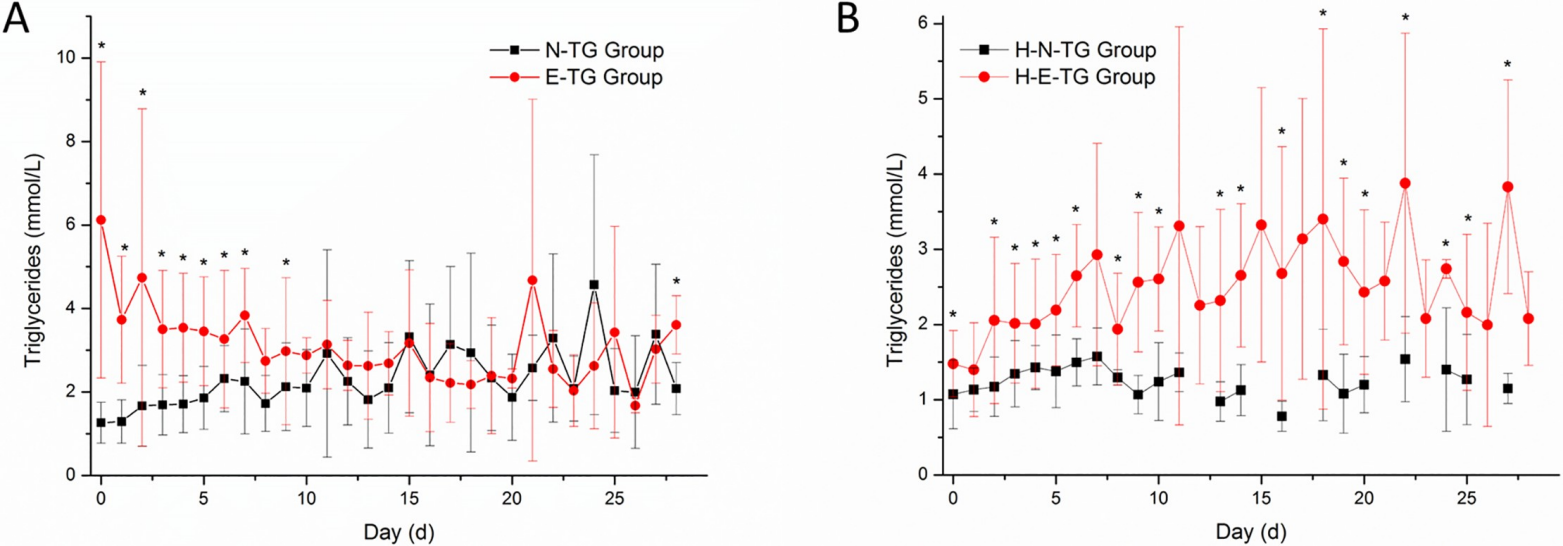
A: Changing trend of serum TG level in N-TG and E-TG group after admission over time. A statistically significant difference was exhibited between N-TG and E-TG group after admission. * p<0.05. B: Changing trend of serum TG level in H-N-TG and H-E-TG group after admission over time. A statistically significant difference was exhibited between H-N-TG and H-E-TG group after admission. * p<0.05.

In total, 40.40% (40 out of 99) of the patients with SAP had an elevated TG level at the time of ICU admission (Fig 1). No significant differences in demographic data were found between the N-TG group and E-TG group, except in pancreatitis aetiology (Table 1). The serum TG changing levels in N-TG and E-TG patients after ICU admission are shown in Fig 3A. Patients in E-TG group at admission had a significantly higher serum TG level within 8 days after admission compared with those in N-TG group. In addition, ICU and 28-day mortality rates seems lower in E-TG group compared with patients in N-TG group, without significant difference (Table 1, S1 Fig). Furthermore, in patients with non-hypertriglyceridaemia-induced SAP, elevated TG level at admission was also not significantly associated with increased mortality (S2 Table).

In-hospital TG elevation events occurred in 47.46% (28/59) of patients in the cohort (Fig 1). The serum TG levels in both H-N-TG and H-E-TG group during their ICU stay are shown in Fig 3B. Table 2 and S3 Table show that patients who have elevated TG level had higher Ranson’s scores at admission, and mean serum TG levels in these patients were significantly higher than in patients who did not experience TG elevation in hospital. During the entire study period, TG elevation obtained in the hospital was directly associated with a higher mortality and a longer residence time in the ICU (Table 2). Moreover, there was a significant difference in mortality at 28 days between patients in H-N-TG group and H-E-TG group (S2 Fig). Results from the propensity score analysis and logistic analysis suggested that TG elevation in hospital is associated with worse outcome (Table 3 and Table 4).

**Table 2:** Demographics, clinical and outcome data of N-TG patient cohort.

Parameters	All (n = 59)	H-N-TG Group (n = 31)	H-E-TG group (n = 28)	P
Demographic Data				
Age, mean (SD),y	50.92 (15.20)	49.55 (17.64)	52.43 (12.10)	0.056
Male, n (%)	36 (61.02)	21 (67.74)	15 (53.57)	0.265
Etiology, n (%)				0.963
Gallstone	36 (61.02)	19 (61.29)	17 (60.71)	
Others	23 (38.98)	12 (38.71)	11 (39.29)	
Underlying medical conditions				
Diabetes Mellitus, n (%)	12 (20.34)	4 (12.90)	8 (28.57)	0.135
Hypertension, n (%)	15 (25.40)			0.945
APACHEⅡScore*, mean (SD)	15.85 (8.58)	13.48 (7.60)	18.25 (8.98)	0.191
Ranson’s Score*, mean (SD)	1.93 (1.13)	1.71 (1.07)	2.18 (1.16)	0.033
TG, mean (SD), mmol/L	1.26 (2.49)	1.08 (0.45)	1.48 (0.44)	<0.001
TG, mean (SD), mg/dL	111.51 (220.36)	95.58 (39.83)	130.98 (38.94)	<0.001
CHOL, mean (SD), mmol/L	1.88 (0.64)	1.88 (0.75)	1.87 (0.51)	<0.001
HDL-C, mean (SD), mmol/L	0.35 (0.21)	0.43 (0.23)	0.26 (0.14)	0.165
LDL-C, mean (SD), mmol/L	0.84 (0.50)	0.96 (0.55)	0.70 (0.40)	0.203
Outcome				
Mechanical Ventilation free days^#^, mean (SD), d	20.07 (8.17)	20.45 (8.59)	19.64 (7.82)	0.052
Renal replacement therapy, n (%)	5 (8.50)	0 (0.00)	5 (17.90)	0.013
Time to start enteral nutrition, mean (SD), d	1.57 (2.15)	1.74 (2.16)	1.42 (2.19)	0.613
Time for parenteral nutrition^#^, mean (SD), d	8.19 (7.63)	8.45 (8.37)	7.89 (6.87)	0.782
28-day mortality, n (%)	15 (75.00)	4 (12.90)	11 (39.29)	0.011
In hospital mortality, n (%)	19 (32.20)	5 (16.13)	14 (50.00)	0.005
ICU LOS, mean (SD),d	14.32 (12.36)	9.33 (9.79)	19.80 (12.73)	0.314

HTG: Hypertriglyceridaemia; APACHE: Acute Physiology and Chronic Health Evaluation; RBC: Red blood cell, HGB: Hemoglobin, HCT: Hematocrit, MCV: Mean corpuscular volume, MCH: Mean corpuscular hemoglobin, MCHC: Mean corpuscular hemoglobin concentration, RDW: Red blood cell distribution width, PLT: Platelets, WBC: white blood cell; INR: International normalized ratio, PT: prothrombin time, APTT: Activated partial thromboplastin time, Fib: fibrinogen, TT: Thrombin time, PaO2: Arterial oxygen partial pressure, PaCO2: Arterial carbon dioxide partial pressure, BE: Base excess, Lac: lactate, PCT: Procalcitonin, TB: Total bilirubin, DB: Direct bilirubin, ALT: Alanine Aminotransferase, AST: Aspartate Aminotransferase, ALP: Alkaline Phosphatase, TP: Total protein, ALB: Albumin, Glu: Glucose, Cys-c: Cystatin C, GGT: Gamma-Glutamyl Transferase, TG: Triglycerides, CHOL: cholesterol, HDL-C: High-density lipoprotein cholesterol, LDL-C: Low-density lipoprotein cholesterol, H/L: HDL-C/ LDL-C, LOS: length of stay; IQR: interquartile range

*on admission

^#^total amount during ICU stay

**Table 3:** Propensity score matching analysis of N-TG SAP patient cohort.

Parameters	All* (n = 30)	H-N-TG group* (n = 15)	H-E-TG group* (n = 15)	P
Demographic Data				
Age, mean (SD),y	49.03 (15.45)	46.47 (17.39)	51.60 (13.34)	0.372
Male, n (%)	18 (60.00)	9 (60.00)	9 (60.00)	>0.999
Etiology, n (%)				0.285
Gallstone	19 (63.33)	10 (66.67)	9 (60.00)	
Alcoholic	0 (0.00)	0 (0.00)	0 (0.00)	
Others	11 (36.67)	5 (33.33)	6 (40.00)	
Underlying medical conditions				
Hypertension	7 (23.33)	5 (33.33)	2 (13.33)	0.208
Diabetes Mellitus, n (%)	7 (23.33)	3 (20.00)	4 (26.67)	0.679
APACHEⅡScore#, mean (SD)	15.13 (7.48)	15.60 (7.98)	14.67 (7.19)	0.739
Ranson’s Score#, mean (SD)	1.93 (0.98)	1.87 (0.99)	2.00 (1.00)	0.716
TG, mean (SD), mmol/L	1.36 (0.52)	1.13 (0.50)	1.61 (0.42)	0.013
CHOL, mean (SD), mmol/L	1.95 (0.57)	2.00 (0.69)	1.88 (0.44)	0.601
HDL-C, mean (SD), mmol/L	0.35 (0.21)	0.44 (0.23)	0.26 (0.15)	0.002
LDL-C, mean (SD), mmol/L	0.86 (0.44)	0.99 (0.50)	0.72 (0.33)	0.121
Mechanical Ventilation free days**, mean (SD), d	20.13 (8.74)	21.80 (7.76)	18.47 (9.58)	0.304
Renal replacement therapy, n (%)	1 (3.33)	0 (0.00)	1 (6.67)	0.326
Time to start enteral nutrition, mean (SD), d	1.36 (1.73)	1.17 (1.99)	1.54 (1.51)	0.602
Time for parenteral nutrition, mean (SD), d	9.77 (8.46)	10.07 (8.85)	9.47 (8.34)	0.850
28-day mortality, n (%)	7 (23.33)	1 (6.67)	6 (40.00)	0.031
In hospital mortality, n (%)	7 (23.33)	2 (13.33)	9 (60.00)	0.008
ICU LOS, mean (SD),d	16.12 (13.77)	12.79 (13.50)	19.64 (13.64)	0.193

APACHE: Acute Physiology and Chronic Health Evaluation; TG: Triglycerides, CHOL: cholesterol, HDL-C: High-density lipoprotein cholesterol, LDL-C: Low-density lipoprotein cholesterol, LOS: length of stay

*Propensity score matching cohort was generated using age, gender, etiology, Underlying medical conditions, APACHEⅡScore, and anson’s Score

#on admission

**within 28 days

**Table 4:** Logistic regression analysis of risk factors for mortality in N-TG group patients.

Variables	Odds Ratio	95% CI		P value
		lower	upper	
Age	1.103	0.922	1.320	0.282
Gender	0.238	0.036	1.572	0.136
APACHEⅡScore	1.041	0.956	1.133	0.357
Ranson’s Score	1.542	0.613	3.877	0.357
Occurrence of TG elevation events in hospitalization	1.354	1.133	1.617	0.001

95% CI: 95% confidence interval

To determine why patients with normal TG level at admission developed new-onset TG elevation events, we analysed treatments that may have induced TG elevation during the ICU stay. Table 5 shows that the amount of propofol usage between the two groups was significantly different. Logistic regression analysis using factors with significantly differences between the H-N-TG group and H-E-TG group was suggested that propofol usage was independently associated with the incidence of hospital-acquired TG elevation (Table 6).

**Table 5:** Treatments which may have influenced Serum triglyceride level during ICU stay in SAP patient cohort with normal TG level at admission.

Parameters^#^	H-E-TG Group (n = 28)	H-N-TG Group (n = 31)	P
Propofol, mean (SD), mg	4907.06 (6696.13)	1794.00 (2404.82)	0.029
Midazolam, mean (SD), mg	914.50 (1451.62)	366.03 (589.14)	0.126
Compound Acid Injection, mean (SD), mL	3159.52 (3231.89)	3815.38 (7031.07)	0.695
Ensure, mean (SD), g	1558.21 (4078.06)	2087.74 (3147.67)	0.783
Peptamen, mean (SD), g	3810.00 (3471.25)	3471.25 (6877.97)	0.801
Crestor, mean (SD), mg	5040.00 (6860.82)	17761.67 (27376.38)	0.294
Insulin, mean (SD), U	514.59 (508.68)	246.68 (373.59)	0.066
Intralipid 20%, mean (SD),mL	928.47 (818.97)	2944.44 (3807.75)	0.193
KabivenTM, mean (SD), mL	4320.00(5176.61)	3280.00 (3485.72)	0.503

HTG: Hypertriglyceridaemia

^#^total amount during ICU stay

**Table 6:** Logistic regression analysis of risk factors for developing TG elevation in N-TG patient group.

Variables	Odds Ratio	95% CI		P value
		lower	upper	
Age	0.993	0.949	1.039	0.751
Gender	0.421	0.847	1.072	0.953
Ranson’s score at admission	3.209	0.856	12.031	0.084
TG at admission	28.604	0.883	926.499	0.059
CHOL at admission	1.358	0.193	9.578	0.759
Propofol usage	11.053	2.324	52.571	0.014

95% CI: 95% confidence interval

## Discussion

The principal findings of this study were that TG elevation event was common in patients admitted to the ICU with a diagnosis of SAP. Hospital-acquired new-onset TG elevation event, was associated with worse outcomes and a longer ICU length of stay. Risk factor analysis indicated that the amount of propofol usage is an independent risk factor for hospital-acquired TG elevation in patients with normal TG level at admission. As far as we know, this is the first study that studies the epidemiology of TG elevation acquired in the hospital and its possible impact on the adverse outcomes of a SAP patients group.

The pancreas is a secretory organ with both endocrine and exocrine functions that is directly involved in lipid metabolism. The presence of free fatty acids in the duodenum causes the release of the peptide hormone pancreazymin-cholecystokinin, which induces the gallbladder to release bile salts and the pancreas to release pancreatic lipase [13, 14]. Pancreatic lipase hydrolyses TG to generate free fatty acids and 2-monoglycerides, which are absorbed by the intestinal mucosa cells [10]. The concurrent pancreatic inflammation and lipid disturbance that occur during an SAP episode, which has been defined by the Atlanta Symposium as acute pancreatitis associated with organ dysfunction or local or regional complications, has drawn much attention in recent years [14–17]. It is well known that hypertriglyceridaemia is one of the leading causes of SAP, after alcohol consumption and gallstones. Although the mechanism of hypertriglyceridaemia -induced SAP is still unclear, the most accepted theory involves accumulation of free fatty acids in the pancreas, which results from excess TG being hydrolysed by pancreatic lipase [18]. However, as mentioned above, earlier studies have focused on the role of SAP in triggering HTG. The dynamic changes in serum TG levels that occur during SAP episodes, especially during an ICU stay, are poorly understood. As parenteral nutrition support is indicated in those patients who are unable to tolerate targeted requirements by the enteral route, and lipids are essential for an efficient source of calories during parenteral nutrition support, it is necessary to evaluate the effect of hospital-acquired TG elevation on patients’ outcomes to improve clinical practice.

Our study characterised demographic data associated with elevated TG level at admission and acquired at ICU stay in patients with SAP. Patients with TG elevation when admitted to ICU had higher severity scores and were younger than patients those without. Thrombocytopenia, electrolyte disorders, and high creatinine levels were highly prevalent in patients with TG elevation at admission. While, patients with hospital-acquired TG elevation had a higher Ranson’s score and were older than those with TG elevation at admission.

An elevated TG level at admission was not associated with increased mortality in our cohort, which is in accord with the findings of a recent meta-analysis [19]. In addition, TG elevation at the time of admission to the ICU was not related to mortality in patients with non-hyperlipidaemia-induced pancreatitis. In China, the prevalence of hypertriglyceridaemia has increased recently because of changing eating habits, an increase in sedentary lifestyles, rising obesity levels and concomitant diabetes mellitus. Hypertriglyceridaemia-induced pancreatitis is often correlated with higher severity and elevated complication rates [20]. Although TG plays an active role in pathogenesis, our study raises the possibility that proinflammatory cytokines and adhesion molecules other than TG are the main determinants of mortality in the early phase of the disease.

We found that hospital-acquired new-onset TG elevation is associated with mortality. All the patients in N-TG group are non-hyperlipidaemia-induced pancreatitis which ensure the comparability of the study. The elevated TG levels observed in SAP patients with a normal TG level at admission could occur for several reasons. Establishing enteral nutrition in these patients is relatively difficult because of persistent intestinal dysfunction, as well as SAP-related complications such as gastrointestinal fistula. Inadequate evaluation of lipid needs during parenteral nutrition may result in TG elevation. Moreover, current guidelines recommend maintaining TG values below 12 mmol/L instead of within the normal range, so clinicians may be poorly informed about lipid control. In addition, accurately determining an individual patient’s need for lipids and proteins is extremely difficult in current clinical practice. Another reason for the occurrence of hospital-acquired TG elevation was prolonged sedation with propofol in patients with SAP who underwent mechanical ventilation. Our study indicates that propofol, not parenteral nutrition, was the main cause of hospital-acquired new-onset TG elevation. These results suggest that midazolam should be used instead of propofol to sedate patients with SAP, to avoid disturbing lipid metabolism [21]. Future studies should focus on interventions to prevent TG elevation happened in the ICU stay, which may directly improve mortality in patients with SAP.

In our study, we defined TG elevation as a serum TG level greater than 1.70 mmol/L. Indeed, TG elevation is usually a biochemical diagnosis that is made on the basis of whether the fasting plasma TG concentration is above a certain cut-off point [22]. Proposed TG elevation definitions vary [14, 23]. For example, the Adult Treatment Panel III guidelines of the National Cholesterol Education Program has proposed four different categories and TG elevation was defined as serum TG level > 150 mg/dL (1.7 mmol/L) [24]. TG elevation severity level has been proposed by another system raised by the Endocrine Society with five clinical strata [25]. No single scheme has become predominant in the clinic although other systems have been proposed. The TG cut-off level is defined mainly on the basis of the long-term risk of cardiovascular disease. In our study, a cut-off value of 1.70 mmol/L was used to define TG elevation, although the inflammation that occurs during SAP and that in patients with cardiovascular disease is obviously different.

This study has some limitations which have to be pointed out. First, this study was retrospective and observation bias is a concern although data were from a prospectively collected database. Second, we only investigated the serum TG levels for 28 days after ICU admission, and we did not have TG records for all patients in our hospital database. We did not include the mean weight/BMI in the demographics as this may also influence the TG levels in patients. The lack of follow-up data prevents our analysis of out-of-hospital outcomes.

To conclude, we retrospectively investigated the incidence, outcome, and risk factors for TG elevation in SAP patients admitted to the ICU. We found that a high incidence of hospital-acquired TG elevation in SAP patients admitted to the ICU. The TG elevation acquired during the ICU stay was associated with worse outcomes and long-term hospitalization of the ICU. Propofol usage was independently associated with the TG elevation occurrence in the ICU.

## Supporting information

S1 Table Demographics, clinical and outcome data of severe acute pancreatitis patient cohort. (DOCX)

S2 Table Comparison among non-hyperlipidemia induced severe acute pancreatitis patient cohort. (DOCX)

S3 Table Demographics, clinical and outcome data of N-TG patient cohort. (DOCX)

S1 Fig Survival analysis. No significant difference in mortality was observed between E-TG group patients and N-TG group patients.
(EMF)

S2 Fig Survival analysis. A significant difference in mortality was observed between H-E-TG patients and H-N-TG patients.
(EMF)

S1 Minimal dataset The minimal anonymized data set necessary to replicate your study. (ZIP)
