# Supplementary material for: Elevated triglycerides level in hospital stay as a risk factor of mortality in patients with severe acute pancreatitis
Source: PLoS One. 2018 Nov 29;13(11):e0207875. doi: 10.1371/journal.pone.0207875 (PMC6264831; doi:10.1371/journal.pone.0207875)
Supplement: S1 Table — (DOCX) [file pone.0207875.s001.docx]

**S1 Table. Demographics, Clinical and Outcome data of severe acute pancreatitis patient cohort**

| **Parameters** | **All**  **(n=99)** | **N-TG Group**  **(n=59)** | **E-TG Group**  **(n=40)** | **P** |
| --- | --- | --- | --- | --- |
| Complete blood count* |  |  |  |  |
| RBC, mean (SD),×10^9^/L | 3.34 (0.75) | 3.25 (0.73) | 3.49 (0.75) | 0.129 |
| HGB, mean (SD), g/L | 99.25 (23.36) | 94.53 (20.73) | 106.26 (25.48) | 0.015 |
| HCT, mean (SD), L/L | 0.31 (0.07) | 0.29 (0.58) | 0.33 (0.72) | 0.008 |
| MCV, mean (SD), fL | 91.40 (6.36) | 89.95 (6.85) | 93.56 (4.86) | 0.005 |
| MCH, mean (SD), pg | 29.68 (2.14) | 29.23 (2.28) | 30.36 (1.73) | 0.010 |
| MCHC, mean (SD), g/L | 272.77 (132.61) | 228.22 (139.21) | 264.42 (120.62) | 0.189 |
| PLT, mean (SD),×10^9^/L | 185.68 (129.94) | 211.72 (132.93) | 146.95 (116.51) | 0.015 |
| WBC, mean (SD),×10^9^/L | 13.90 (7.92) | 14.54 (8.52) | 12.96 (7.12) | 0.338 |
| Coagulation test* |  |  |  |  |
| INR, mean (SD) | 1.33 (0.32) | 1.35 (0.26) | 1.28 (0.38) | 0.298 |
| PT, mean (SD), s | 15.54 (3.61) | 15.87 (2.98) | 15.06 (4.37) | 0.286 |
| APTT, mean (SD), s | 42.59 (18.09) | 47.21 (20.92) | 35.80 (9.59) | 0.002 |
| Fib, mean (SD), g/L | 3.75 (2.05) | 2.87 (1.49) | 5.05 (2.09) | <0.001 |
| TT, mean (SD), s | 19.28 (7.20) | 19.40 (7.03) | 19.09 (7.54) | 0.841 |
| Arterial Blood Gas Test* |  |  |  |  |
| pH, mean (SD) | 7.33 (0.31) | 7.37 (0.19) | 7.28 (0.42) | 0.164 |
| PaO2, mean (SD), mmHg | 103.85 (42.33) | 113.55 (45.49) | 88.08 (31.27) | 0.007 |
| PaCO2, mean (SD), mmHg | 35.75 (8.09) | 35.53 (9.13) | 36.10 (6.17) | 0.755 |
| BE, mean (SD), mmol/L | -3.07 (4.35) | -2.68 (4.30) | -3.72 (4.44) | 0.291 |
| Lac, mean (SD), mmol/L | 2.31 (2.35) | 2.46 (2.85) | 2.07 (1.23) | 0.468 |
| Biochemical analysis* |  |  |  |  |
| PCT, mean (SD), ng/mL | 9.53 (16.83) | 8.70 (18.55) | 10.63 (14.46) | 0.634 |
| Serum sodium, mean (SD), mmol/L | 138.10 (6.58) | 137.80 (7.16) | 138.54 (5.70) | 0.586 |
| Serum potassium, mean (SD), mmol/L | 4.11 (0.63) | 4.16 (0.67) | 4.03 (0.57) | 0.297 |
| Serum chloride, mean (SD), mmol/L | 108.11 (6.57) | 106.98 (6.65) | 109.78 (6.16) | 0.039 |
| TB, mean (SD), mmol/L | 35.23 (52.54) | 29.78 (32.17) | 42.88 (71.84) | 0.230 |
| DB, mean (SD), mmol/L | 27.52 (49.81) | 21.52 (26.35) | 36.38 (71.19) | 0.146 |
| ALT, mean (SD), mmol/L | 97.16 (303.71) | 92.96 (308.88) | 103.03 (300.12) | 0.874 |
| AST, mean (SD), mmol/L | 183.71 (711.45) | 112.08 (324.72) | 289.35 (1046.84) | 0.226 |
| ALP, mean (SD), mmol/L | 105.91 (148.03) | 125.14 (187.24) | 78.98 (51.57) | 0.133 |
| TP, mean (SD), mmol/L | 46.17 (9.88) | 43.76 (10.50) | 49.74 (7.72) | 0.003 |
| ALB, mean (SD), mmol/L | 25.53 (7.06) | 23.44 (6.84) | 28.61 (6.26) | <0.001 |
| Glu, mean (SD), mmol/L | 10.52 (3.47) | 9.80 (3.22) | 11.58 (3.58) | 0.011 |
| Creatinine, mean (SD), mmol/L | 143.18 (169.33) | 100.53 (128.34) | 205.03 (201.52) | 0.002 |
| Cys-c, mean (SD), mmol/L | 1.37 (1.02) | 1.21 (0.78) | 1.61 (1.26) | 0.058 |
| GGT, mean (SD), mmol/L | 94.26 (117.49) | 105.86 (138.21) | 77.43 (76.98) | 0.241 |

HTG: Hypertriglyceridaemia; RBC: Red blood cell, HGB: Hemoglobin, HCT: Hematocrit, MCV: Mean corpuscular volume, MCH: Mean corpuscular hemoglobin, MCHC: Mean corpuscular hemoglobin concentration, RDW: Red blood cell distribution width, PLT: Platelets, WBC: white blood cell; INR: International normalized ratio, PT: prothrombin time, APTT: Activated partial thromboplastin time, Fib: fibrinogen, TT: Thrombin time, PaO2: Arterial oxygen partial pressure, PaCO2: Arterial carbon dioxide partial pressure, BE: Base excess, Lac: lactate, PCT: Procalcitonin, TB: Total bilirubin, DB: Direct bilirubin, ALT: Alanine Aminotransferase, AST: Aspartate Aminotransferase, ALP: Alkaline Phosphatase, TP: Total protein, ALB: Albumin, Glu: Glucose, Cys-c: Cystatin C, GGT: Gamma-Glutamyl Transferase, *on admission
