# Supplementary material for: Elevated triglycerides level in hospital stay as a risk factor of mortality in patients with severe acute pancreatitis
Source: PLoS One. 2018 Nov 29;13(11):e0207875. doi: 10.1371/journal.pone.0207875 (PMC6264831; doi:10.1371/journal.pone.0207875)
Supplement: S2 Table — (DOCX) [file pone.0207875.s002.docx]

**S2 Table. Comparison among non-hyperlipidemia induced severe acute pancreatitis patient cohort**

| **Parameters** | **All**  **(n=80)** | **N-TG Group**  **(n=59)** | **E-TG Group**  **(n=40)** | **P** |
| --- | --- | --- | --- | --- |
| Demographic Data |  |  |  |  |
| Age, mean (SD),y | 49.54 (14.51) | 50.92 (15.20) | 45.67 (11.82) | 0.156 |
| Male, n (%) | 51 (63.80) | 36 (61.02) | 15 (71.4) | 0.400 |
| Etiology, n (%) |  |  |  | 0.139 |
| Biliary | 43 (43.43) | 36 (61.02) | 7 (17.50) |  |
| Alcoholic | 6 (6.06) | 0 (0.00) | 6 (15.00) |  |
| Others | 31 (31.32) | 23 (38.98) | 8 (20.00) |  |
| Underlying medical conditions |  |  |  |  |
| Hypertension | 19 (23.80) | 15 (25.40) | 4 (19.00) | 0.561 |
| Diabetes Mellitus, n (%) | 16 (20.00) | 12 (20.34) | 4 (19.00) | 0.900 |
| APACHEⅡScore*, mean (SD) | 15.70 (7.84) | 15.85 (8.58) | 15.53 (5.36) | 0.818 |
| Ranson’s Score*, mean (SD) | 2.05 (1.16) | 1.93 (1.13) | 2.38 (1.20) | 0.128 |
| TG, mean (SD), mmol/L | 1.87 (1.16) | 1.26 (0.48) | 3.52 (0.78) | <0.001 |
| CHOL, mean (SD), mmol/L | 2.35 (1.18) | 1.86 (0.64) | 3.62 (1.36) | <0.001 |
| HDL-C, mean (SD), mmol/L | 0.35 (0.26) | 0.35 (0.21) | 0.37 (0.38) | 0.726 |
| LDL-C, mean (SD), mmol/L | 0.96 (0.65) | 0.84 (0.50) | 1.29 (0.88) | 0.006 |
| Mechanical Ventilation free days^#^, mean (SD), d | 19.90 (8.57) | 20.07 (8.17) | 19.43 (9.78) | 0.771 |
| Renal replacement therapy, n (%) | 7 (8.8) | 5 (8.50) | 2 (9.50) | 0.886 |
| Time to start enteral nutrition, mean (SD), d | 1.95 (2.90) | 1.57 (2.15) | 3.00 (4.27) | 0.083 |
| Time for parenteral nutrition^#^, mean (SD), d | 9.14 (7.99) | 8.19 (7.63) | 11.81 (8.57) | 0.074 |
| 28-day mortality, n (%) | 18 (22.50) | 15 (25.42) | 3 (14.30) | 0.300 |
| In hospital mortality, n (%) | 24 (30.00) | 19 (32.20) | 5 (23.80) | 0.477 |
| ICU LOS, mean (SD),d | 15.44 (13.88) | 14.32 (12.36) | 20.35 (19.91) | 0.243 |

HTG: Hypertriglyceridaemia; APACHE: Acute Physiology and Chronic Health Evaluation; TG: Triglycerides, CHOL: cholesterol, HDL-C: High-density lipoprotein cholesterol, LDL-C: Low-density lipoprotein cholesterol, H/L: HDL-C/ LDL-C, LOS: length of stay; IQR: interquartile range

*on admission

^#^within 28 days
