# Supplementary material for: Elevated triglycerides level in hospital stay as a risk factor of mortality in patients with severe acute pancreatitis
Source: PLoS One. 2018 Nov 29;13(11):e0207875. doi: 10.1371/journal.pone.0207875 (PMC6264831; doi:10.1371/journal.pone.0207875)
Supplement: S3 Table — (DOCX) [file pone.0207875.s003.docx]

**S3 Table. Demographics, Clinical and Outcome data of N-TG patient cohort**

| **Parameters** | **All**  **(n=59)** | **H-N-TG group**  **(n=31)** | **H-E-TG Group**  **(n=28)** | **P** |
| --- | --- | --- | --- | --- |
| Complete blood count* |  |  |  |  |
| RBC, mean (SD),×10^9^/L | 3.25 (0.73) | 3.45 (0.82) | 3.04 (0.57) | 0.129 |
| HGB, mean (SD), g/L | 94.53 (20.73) | 99.93 (21.70) | 88.75 (18.29) | 0.015 |
| HCT, mean (SD), L/L | 0.29 (0.58) | 0.31 (0.06) | 0.27 (0.05) | 0.008 |
| MCV, mean (SD), fL | 89.95 (6.85) | 89.67 (7.83) | 90.25 (5.76) | 0.005 |
| MCH, mean (SD), pg | 29.23 (2.28) | 29.28 (2.58) | 29.16 (1.95) | 0.010 |
| MCHC, mean (SD), g/L | 228.22 (139.21) | 188.14 (149.69) | 271.15 (114.67) | 0.189 |
| PLT, mean (SD),×10^9^/L | 211.72 (132.93) | 194.77 (134.10) | 229.89 (131.62) | 0.015 |
| WBC, mean (SD),×10^9^/L | 14.54 (8.52) | 12.62 (7.00) | 16.59 (9.40) | 0.338 |
| Coagulation test* |  |  |  |  |
| INR, mean (SD) | 1.35 (0.26) | 1.37 (0.29) | 1.34 (0.21) | 0.298 |
| PT, mean (SD), s | 15.87 (2.98) | 16.03 (3.43) | 15.69 (2.42) | 0.286 |
| APTT, mean (SD), s | 47.21 (20.92) | 49.08 (26.49) | 45.05 (11.81) | 0.002 |
| Fib, mean (SD), g/L | 2.87 (1.49) | 2.80 (1.64) | 2.95 (1.29) | <0.001 |
| TT, mean (SD), s | 19.40 (7.03) | 20.31 (9.30) | 18.31 (2.13) | 0.841 |
| Arterial Blood Gas Test* |  |  |  |  |
| pH, mean (SD) | 7.37 (0.19) | 7.35 (0.26) | 7.39 (0.06) | 0.164 |
| PaO2, mean (SD), mmHg | 113.55 (45.49) | 116.02 (50.74) | 111.10 (40.43) | 0.007 |
| PaCO2, mean (SD), mmHg | 35.53 (9.13) | 34.56 (9.46) | 36.49 (8.86) | 0.755 |
| BE, mean (SD), mmol/L | -2.68 (4.30) | -3.37 (3.67) | -1.98 (4.81) | 0.291 |
| Lac, mean (SD), mmol/L | 2.46 (2.85) | 3.20 (3.85) | 1.71 (0.75) | 0.468 |
| Biochemical analysis* |  |  |  |  |
| PCT, mean (SD), ng/mL | 8.70 (18.55) | 13.64 (25.81) | 4.84 (8.69) | 0.634 |
| Peak TG level prior to ICU admission, mean (SD), mmol/L | 1.26 (0.56) | 1.05 (0.86) | 1.33 (0.99) | 0.482 |
| Serum sodium, mean (SD), mmol/L | 137.80 (7.16) | 137.32 (6.72) | 138.31 (7.70) | 0.586 |
| Serum potassium, mean (SD), mmol/L | 4.16 (0.67) | 4.11 (0.66) | 4.21 (0.68) | 0.297 |
| Serum chloride, mean (SD), mmol/L | 106.98 (6.65) | 107.18 (6.01) | 106.76 (7.38) | 0.039 |
| TB, mean (SD), mmol/L | 29.78 (32.17) | 28.74 (30.04) | 30.97 (35.04) | 0.230 |
| DB, mean (SD), mmol/L | 21.52 (26.35) | 18.48 (20.14) | 24.88 (31.91) | 0.146 |
| ALT, mean (SD), mmol/L | 92.96 (308.88) | 43.37 (75.71) | 150.19 (443.72) | 0.874 |
| AST, mean (SD), mmol/L | 112.08 (324.72) | 57.67 (90.52) | 172.32 (458.52) | 0.226 |
| ALP, mean (SD), mmol/L | 125.14 (187.24) | 88.07 (70.18) | 167.92 (260.51) | 0.133 |
| TP, mean (SD), mmol/L | 43.76 (10.50) | 42.65 (11.43) | 44.99 (9.43) | 0.003 |
| ALB, mean (SD), mmol/L | 23.44 (6.84) | 22.98 (7.17) | 23.94 (6.56) | <0.001 |
| Glu, mean (SD), mmol/L | 9.80 (3.22) | 9.38 (3.58) | 10.26 (2.77) | 0.011 |
| Creatinine, mean (SD), mmol/L | 100.53 (128.34) | 84.30 (105.99) | 117.93 (148.68) | 0.002 |
| Cys-c, mean (SD), mmol/L | 1.21 (0.78) | 1.11 (0.81) | 1.32 (0.75) | 0.058 |
| GGT, mean (SD), mmol/L | 105.86 (138.21) | 85.63 (118.00) | 127.54 (156.29) | 0.241 |

RBC: Red blood cell, HGB: Hemoglobin, HCT: Hematocrit, MCV: Mean corpuscular volume, MCH: Mean corpuscular hemoglobin, MCHC: Mean corpuscular hemoglobin concentration, RDW: Red blood cell distribution width, PLT: Platelets, WBC: white blood cell; INR: International normalized ratio, PT: prothrombin time, APTT: Activated partial thromboplastin time, Fib: fibrinogen, TT: Thrombin time, PaO2: Arterial oxygen partial pressure, PaCO2: Arterial carbon dioxide partial pressure, BE: Base excess, Lac: lactate, PCT: Procalcitonin, TB: Total bilirubin, DB: Direct bilirubin, ALT: Alanine Aminotransferase, AST: Aspartate Aminotransferase, ALP: Alkaline Phosphatase, TP: Total protein, ALB: Albumin, Glu: Glucose, Cys-c: Cystatin C, GGT: Gamma-Glutamyl Transferase,

*on admission
